# Ag^+^ doped into azo-linked conjugated microporous polymer for volatile iodine capture and detection of heavy metal ions

**DOI:** 10.1038/s41598-018-32383-5

**Published:** 2018-09-19

**Authors:** Minghan Liu, Chan Yao, Chunbo Liu, Yanhong Xu

**Affiliations:** 1 Key Laboratory of Preparation and Applications of Environmental Friendly Materials (Jilin Normal University), Ministry of Education, Changchun, 130103 China; 2 Key Laboratory of Functional Materials Physics and Chemistry of the Ministry of Education, Jilin Normal University, Siping, 136000 China; 3 Institute of Green Chemistry & Chemical Technology, Jiangsu University, Zhenjiang, 212013 China

## Abstract

We herein report the construction of a novel azo-linked conjugated microporous polymers (Ag@Azo_TPE_-CMP), which possesses permanent porous structure and Ag^+^ loading up of 7.62% in the skeleton as effective sorption sites. Ag@Azo_TPE_-CMP shows considerable adsorption capacity of iodine of 202 wt% in iodine vapor at 350 K. In addition, Ag@Azo_TPE_-CMP can effectively remove heavy ions from ethanol-water solution.

## Introduction

Conjugated microporous polymers (CMPs) are a unique class of porous organic polymers (POPs), which show a permanent three-dimensional pore structure and extended π-conjugated skeleton. Taking advantage of the large surface areas, microporosity, the large pore volume as well as high thermal and chemical stability, these porous materials are of great important applications in various fields such as gas storage^1–3^, light emitters^4–6^, light-harvesting antennae^7–9^, heterogeneous catalyst^10–14^, super-capacitive energy storage^15–18^, and fluorescent sensors^19,20^, etc. In all of these applications, the porosity of the CMPs is an essential as a class of functional porous medium. Cooper *et al*. have done a lot of works in this point, they utilize the designable flexibility of the conjugated networks of CMPs, making it possible to finely tune the porosity of CMPs by varying the strut length and geometry of monomers^21–23^. Recently, researchers have found that CMPs can be used as effective absorbents for safe and long-term capture and storage of iodine, not only because of their high surface areas, but also owing to containing high affinity binding sites for iodine adsorption^24–28^. In order to enhance the iodine capture ability of CMPs, several strategies have been employed, for example, increasing the surface areas, bearing ionic bond, phenyl ring, and triple-bond, containing enriched π-electron, and introducing heteroatom groups such as N-rich and S-rich groups into the polymers^24–28^. These novel materials have exhibited a high iodine uptake capacity, such as PAF-24-25 (*S*_BET_ = 82–262 m^2^ g^−1^, 260–276 wt.%)^24^, HCMP-3 (*S*_BET_ = 82 m^2^ g^−1^, 276 wt.%)^25^, AzoPPN (*S*_BET_ = 400 m^2^ g^−1^, 290 wt.%)^26^, SCMP-II (*S*_BET_ = 120 m^2^ g^−1^, 345 wt.%)^27^, and SCMP-2 (*S*_BET_ = 855 m^2^ g^−1^, 222 wt.%)^28^.

In the field of iodine adsorption, CMPs has made some impressive progress, but it remains to be developed. It is well-known that the properties of materials were crucially determined by the characters of building units. Azo compounds are one of the most widely used ligands in coordination chemistry^29^, and have been widely used in optics^30^ and drug delivery systems^31^. The obtained azo-porous networks possess outstanding properties: (1) azo-linked porous polymers based on -N=N- bonds are promising iodine adsorbents due to their electron rich pore surface; (2) the surface of CMPs with a lot of polar groups can significantly enhance their iodine binding energy, resulting in enhancement in iodine uptake. In addition, diazo-coupling polymerization of aryl amines with phenols exhibits cost-effective advantages such as cheap catalyst, mild reaction conditions, and high yield used in the polymerization, which are essential for scale-up preparation of porous materials with potential application.

**Figure 1:**
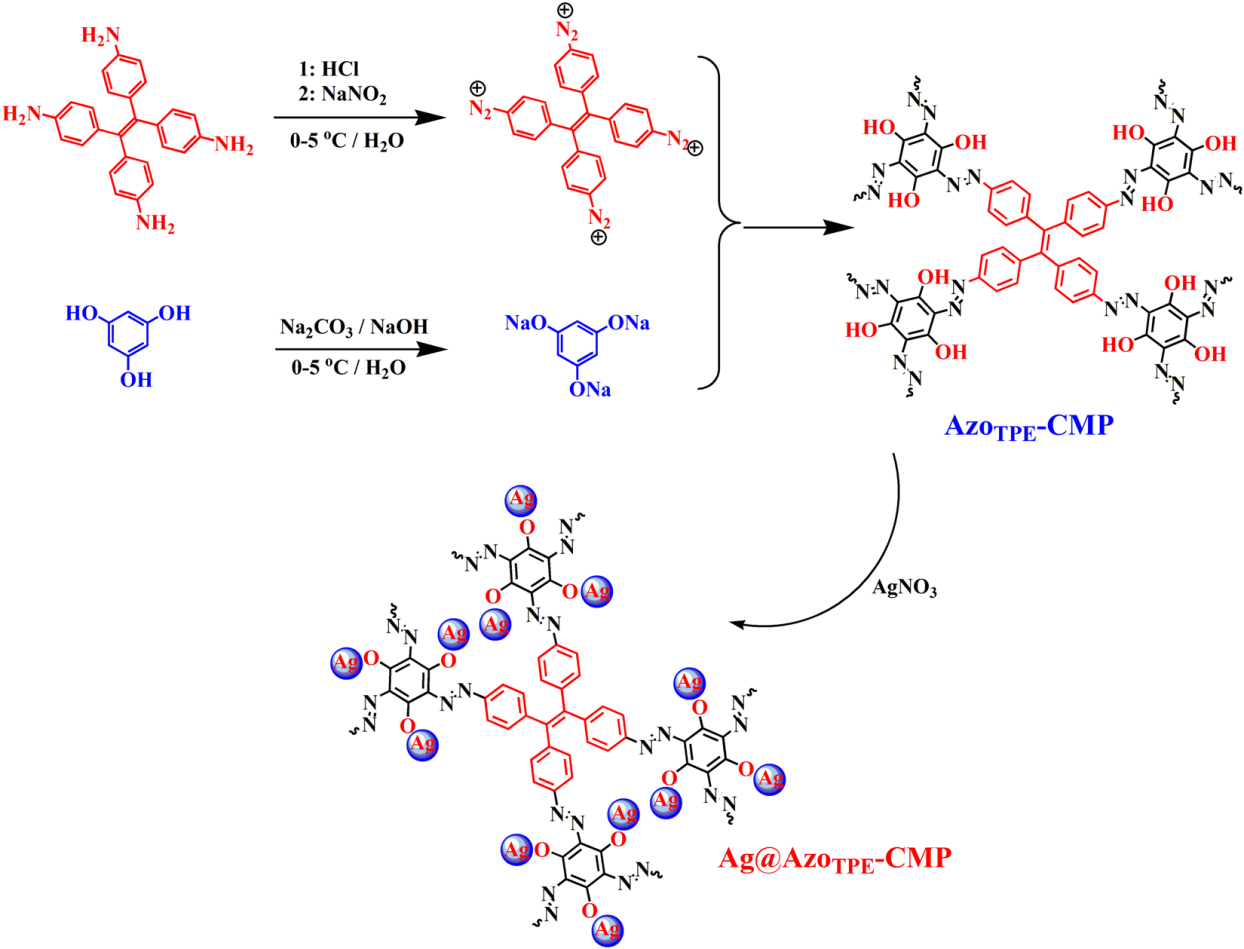
Synthetic routes of AzoTPE-CMP and Ag@AzoTPE-CMP.

Taking into account the advantages above, herein, a novel azo-linked CMP network was synthesized based on diazo-coupling reaction of 4-(1,2,2-tris(4-aminophenyl) vinyl) aniline (TAVA) and 1,3,5-trihydroxybenzen (TDB), which possessed plenty of polar -OH groups on the surface of the skeleton (Azo_TPE_-CMP) (Fig. 1). It is well-known that MOFs loaded with metal silver ions have been proven to be an efficient method for developing new iodine adsorption materials^32^. However, the poor stability of MOFs materials limits their practical applications. In this work, the integration of azo and phenolic -OH gave the ability of the CMP to chelate with metal silver ions, yielding Ag-doped CMP network with Ag^+^ loading up of 7.62% (Ag@Azo_TPE_-CMP). Served as absorbents, the Ag^+^-coordinated CMP exhibits high-speed iodine capture both in vapor and solution, and excellent detection of heavy metal ion. Besides that, the resultant metal silver coordinated CMP showed good stability and recyclability.

## Synthesis and Characterization

Azo_TPE_-CMP network was synthesized by diazo-coupling reaction of 4-(1,2,2-tris(4-aminophenyl) vinyl) aniline (TAVA) and 1,3,5-trihydroxybenzen (TDB). The general synthetic route towards Azo_TPE_-CMP polymer is shown in Fig, 1. The insoluble polymers were filtered and washed with water, tetrahydrofuran, chloroform and methanol, respectively, in order to remove the inorganic salts, organic monomers, residual catalyst, and oligomers.

**Figure 2:**
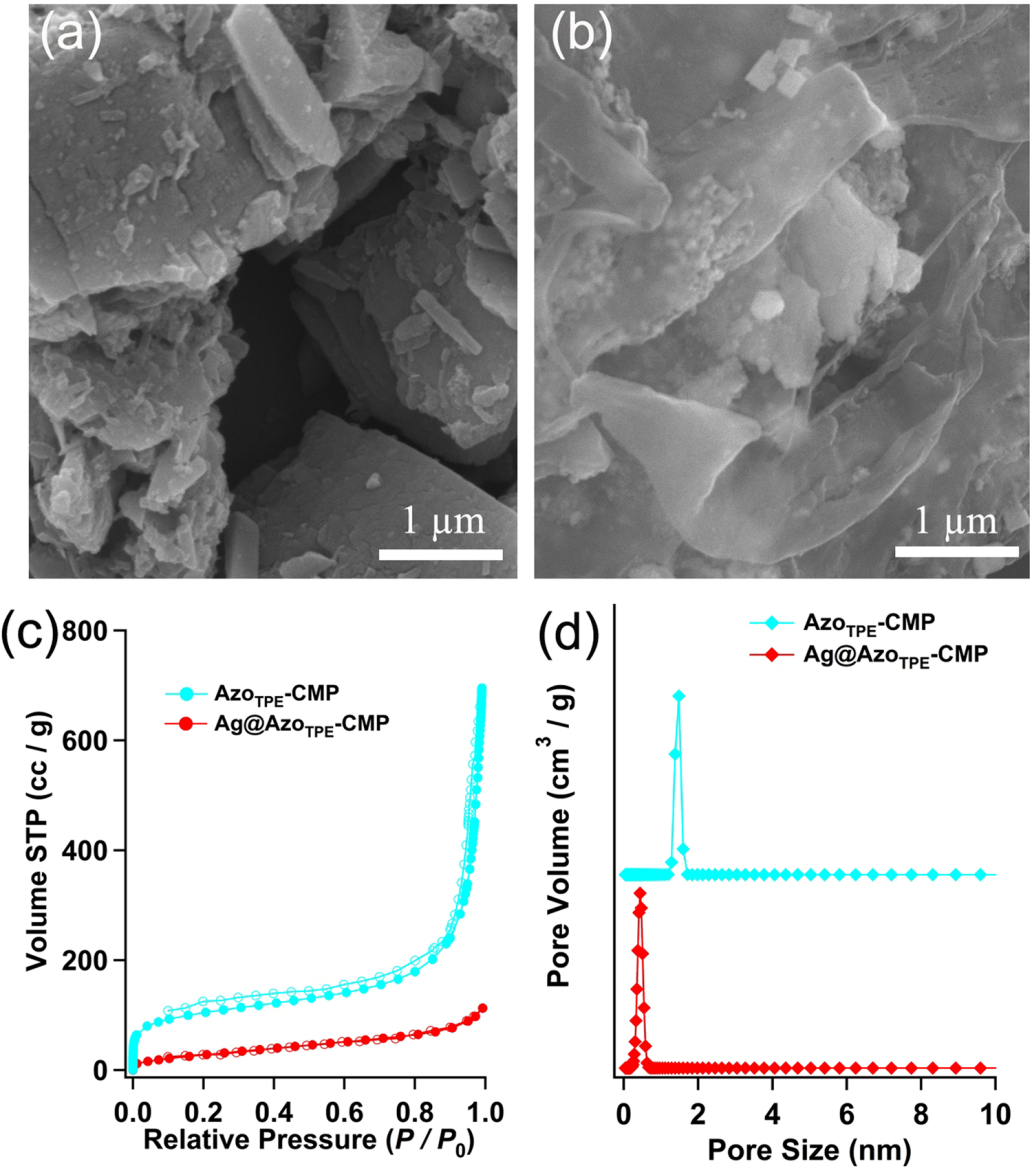
SEM-images for (**a**) Azo_TPE_-CMP, and (**b**) Ag@Azo_TPE_-CMP; (**c**) N_2_ sorption isotherm at 77 K (filled squares: adsorption, open squares: desorption); and (**d**) pore size distribution of the Azo-CMPs.

The Azo_TPE_-CMP was extensively characterized using the solid-state cross-polarization magic angle spinning (CP/MAS) ^13^C NMR, powder X-ray diffraction (PXRD), FT-IR spectroscopy, elemental analysis, scanning electron microscopy (SEM), and thermogravimetric analysis (TGA) (for details, see supporting information). Firstly, the structural integrity of polymers was verified by using CP-MAS ^13^C NMR (Fig. S1). The broad chemical shifts appeared in the region between 120 and 170 ppm is associated with the aromatic carbon atoms of the framework. The peaks located at 108 ppm, which was attributed to the vinyl carbons of TAVA, also verified the successful synthesis of CMP. The broad featureless PXRD patterns of CMPs indicate that the two polymers have amorphous characters (Fig. S2). The structural integrity of CMPs was further verified using FT-IR analysis (Fig. S3). For Azo_TPE_-CMP, the bands around 1650 and 1400 cm^−1^ were attributed to the stretching of C=C and N=N bonds, and the broad peaks around 3430 cm^−1^ were assigned to the Ar-OH groups, confirmed the successful coupling reaction between the monomers. For Ag@Azo_TPE_-CMP, a new peak appeared at 1368 cm^−1^, possibly assigning to the C-O vibration of Ar-OAg, which indicated Ag^+^ was coordinated with phenolic -OH (Fig. S3). The scanning electron microscopy (SEM) analysis was performed in order to investigate the bulk scale morphology of the CMPs (Fig. 2a,b). From SEM images, we can see that the polymers are composed of irregular spherical solids from nanometers to microns. The two azo-linked CMPs were composed of agglomerated plate-shaped particles with a particle size more than 1 micron. Further analysis of the CMPs by TGA showed that the Azo_TPE_-CMP network was thermally stable up to 380 °C under N_2_ atmosphere presumably due to well-spaced charged groups within three-dimensional network. The slight weight loss below 200 °C was mostly associated with the trapped moisture and solvent molecules in the pores (Fig. S4). Compare to Azo_TPE_-CMP, Ag@Azo_TPE_-CMP displayed much higher stability, and its value was as high as 500 °C. Through high-resolution transmission electron microscopy (HR-TEM), we can see that Ag particles have been included successfully for Ag@Azo_TPE_-CMP compared with non-Ag Azo_TPE_-CMP (Fig. S5). Notably, Azo_TPE_-CMP and Ag@Azo_TPE_-CMP polymers showed strong and broad absorption in the UV/Vis adsorption range from 200 to 450 nm (Fig. S6a). Compare to AgNO_3_/Azo_TPE_-CMP complex, the Ag@Azo_TPE_-CMP exhibited a broad absorbance band, also suggesting the coordination between Ag^+^ and phenolic -OH (Fig. S6a). Then, we investigated the luminescence properties of the two CMPs. The Azo_TPE_-CMP was non-emissive, and the Ag@Azo_TPE_-CMP showed the emission band at 402 nm (Fig. S6b).

In addition, X-ray photoelectron spectroscopy (XPS) was performed to explore whether and how the silver coordinated with the porous polymer. Both survey scan and narrow scan (N1s) were performed. C1s (~282.50 ev), O1s (~530.53 ev) and N1s (~400.01 ev) peaks were observed in the XPS spectra of the two samples (Fig. S7). The new peaks at 700–800 eV and 300–400 eV in the O1s XPS spectrum of Ag@Azo_TPE_-CMP may be assigned to -O-Ag, implying that Ag^+^ might coordinate with the phenolic -OH, and AgNO_3_ was not simply physically adsorbed. Moreover, the BE of N1s changed little, implying that -N=N- might not involve coordination with metal ions. Based on the above analysis, it can be deduced that AgNO_3_ should be chelated with Ar-OH to form Ar-O-Ag moieties during the metallization process.

In order to characterize the porosity parameters of azo-linked CMPs, the nitrogen sorption isotherms were measured at 77 K. As shown in Fig. 2c, Azo_TPE_-CMP displays a combination of type-I and IV sorption profiles, according to the IUPAC classification. At low relative pressure, a sharp nitrogen gas uptake reflects the microporous nature of the Azo_TPE_-CMP network. And the nitrogen sorption in the high-pressure region (*P*/*P*_0_ > 0.9) increases with increasing pressure, suggesting a large external surface owing to the loose packing of small particles. The apparent Brunauer-Emmett-Teller (BET) surface areas for the polymers were calculated over the relative pressure range *P*/*P*_0_ = 0.015–0.1, which was found to give a positive value of C in the BET equation. Azo_TPE_-CMP exhibited the BET surface area of 366 m^2^ g^−1^. The total pore volume calculated with nitrogen gas adsorbed at *P*/*P*_0_ = 0.99 was 1.072 cm^3^ g^−1^, and the pore size mainly centered at 1.7 nm, as obtained by the nonlocal density functional theory (NLDFT) (Fig. 2d). Compare to Azo_TPE_-CMP, Ag@Azo_TPE_-CMP showed the lower BET surface area and the total pore volume were 47 m^2^ g^−1^ and 0.110 cm^3^ g^−1^, respectively. In addition, the pore size mainly centered at 0.5 nm. These results indicated that the metal silver ions have been loaded into the pores of the CMP skeleton.

## Discussion

### Iodine capture

**Figure 3:**
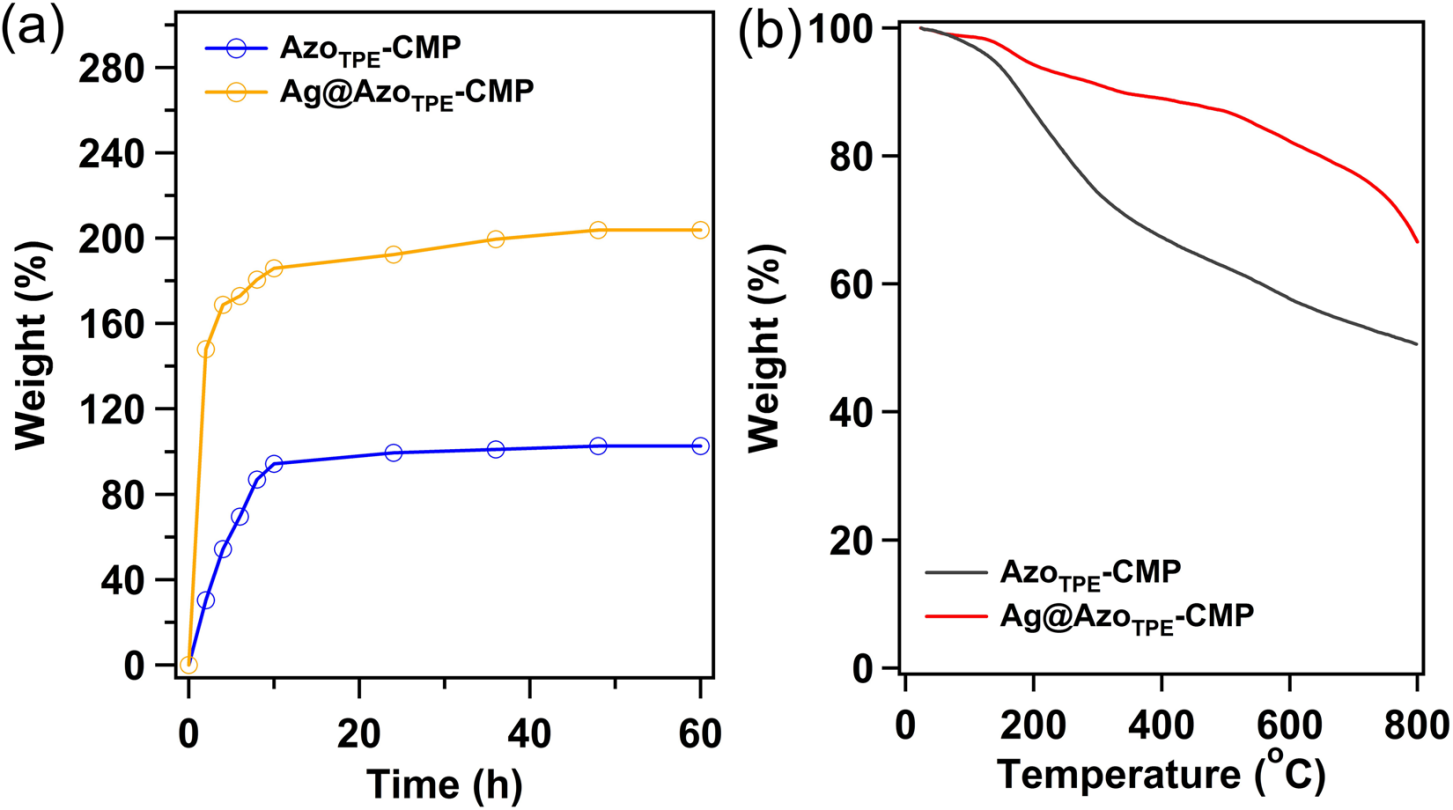
(**a**) Gravimetric uptake of iodine as a function of time at 350 K; (**b**) TGA trace of I_2_ loading azo-linked CMP.

The considerable porous characters, nitrogen- and silver-rich nature of Azo_TPE_-CMP prompted us to assessing their performances for iodine capture. The I_2_ capture process was conducted at 350 K and ambient pressure, which are typical fuel reprocessing conditions. Due to the sample’s color was deep-black, an apparent color change was not observed with time progressed. Figure 3a shows the weight of the CMPs at various time intervals during the iodine uptake. The iodine capture uptake increased significantly with extended contact time. The results suggested that the mass of iodine uptake increased significantly in the initial 10 h and reached a platform thereafter, implying the system basically is saturated after 36 h. The saturated I_2_ loading of Azo_TPE_-CMP and Ag@Azo_TPE_-CMP were measured to be 108 and 202 wt.%, respectively. The thermogravimetric analysis (TGA) of the I_2_-loaded CMP samples reveal a significant weight loss from 90 to 300 °C (Fig. 3b), the calculated iodine mass loss were 122 and 194 wt.% for Azo_TPE_-CMP and Ag@Azo_TPE_-CMP, respectively, which are close to the saturated adsorption value. The I_2_ uptake for Ag@Azo_TPE_-CMP is 1.87-times than that of Azo_TPE_-CMP, which may be attributed to the coordination interaction of silver ions with iodine molecules. X-ray photoelectron spectroscopy (XPS) of the Ag@Azo_TPE_-CMP indicated that the coexistence of elemental iodine and triiodide ions, which suggests a hybrid of physisorption and chemisorption (Fig. S8). However, the XPS of Azo_TPE_-CMP implied that the adsorption of iodine was mainly physisorption. Therefore, Ag@Azo_TPE_-CMP displayed a higher adsorption iodine value than that of Azo_TPE_-CMP. Furthermore, the two samples can be efficiently recycled and reused for five cycles without significant loss of iodine uptake (Fig. S9). At the same time, the addition of loading I_2_ of azo-linked CMP in fresh ethanol could be easily remove the encapsulated iodine from the network. The color of the ethanol solution deepened from colorless to dark brown, which clearly indicates that I_2_ guests are released from the azo-linked networks (Fig. S10).

In addition, the capability of trapping iodine of the two CMP polymers was also tested in solution at ambient conditions. The two samples (30 mg) were immersed in cyclohexane solution of I_2_ in a small sealed flask at room temperature. The purple color of the iodine solution gradually changed from dark purple to light purple and finally to paler (Fig. S11). The UV/vis spectroscopy was used to characterize the adsorption kinetic of iodine (Fig. S12). The UV/Vis absorption intensity of the samples was decreased with the prolonged action time. The adsorption kinetics of iodine at 25 °C were presented, as illustrated in Fig. S13. Two stages of adsorption kinetics of the iodine were observed: the adsorption capacity for iodine increased quickly during the first 8 h, and after that a slowly increased iodine uptake until equilibrium. The adsorption performances of the two CMPs can be best fitted by Langmuir pseudo-first-order kinetic models (Fig. S14), which show the correlation coefficient R^2^ values of 0.9232 and 0.9336 for Azo_TPE_-CMP and Ag@Azo_TPE_-CMP, respectively. Finally, the two polymers exhibited the removal efficiencies of up to 99.9% in the iodine solutions with a concentration of 4 mg mL^−1^, which far exceeded that of functionalized MIL-53-NH_2_ (60%)^33^, metalloporphyrin-based NiP-CMP (56%)^34^. Moreover, the adsorption isotherm is a significant factor in determining the saturated adsorption capacity (Fig. S15). The adsorption plot of equilibrium concentration versus adsorption capacity showed that the two adsorption stages. Firstly, the equilibrium absorption increased linearly with the increase of iodine concentration. Compared with the Freundlich equation, the fitting of Langmuir equation is more in line with the experimental curve, the calculation results suggested that a monolayer adsorption behavior for iodine on the surface of the two samples. The adsorption reached the maximum uptake without relation to the increasing iodine concentration. From the kinetic studies, Azo_TPE_-CMP and Ag@Azo_TPE_-CMP represent a high iodine uptake of 1991 and 2598 mg g^−1^, respectively.

### Detection of heavy metal

**Figure 4:**
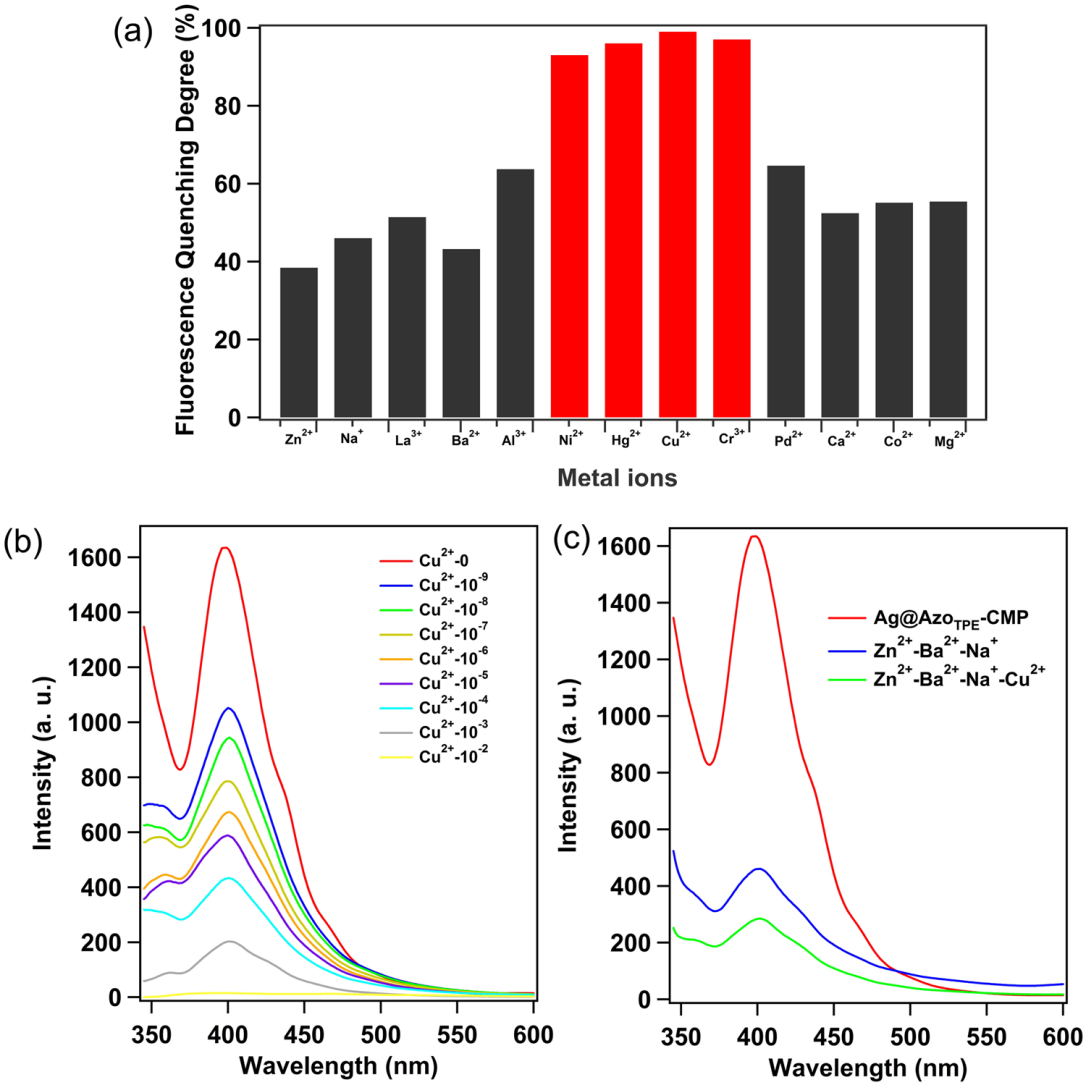
(**a**) Degree of fluorescence quenching of the Ag@Azo_TPE_-CMP in ethanol-water solutions of different metal ions (10^−2^ M). Red bars are for Hg^2+^, Cu^2+^, Cr^3+^, and Ni^2+^. (**b**) The fluorescence intensity of the Ag@Azo_TPE_-CMP in ethanol-water solutions with different concentrations of Cu^2+^. (**c**) The degree of fluorescence quenching of Cu^2+^ (10^−3^ M) in ethanol-water solutions of different metal ions (10^−2^ M).

In recent years, with the rapid development of various industries in the world, the amount of industrial wastewater emissions also showed a sharp upward trend, which makes the water pollution become more and more serious, and the task of wastewater management needs to be carried out urgently. Heavy metal wastewater is considered to be one of the most serious industrial wastes endangering the environment and human health. In this work, the Ag@Azo_TPE_-CMP was found to be capable of efficiently detecting heavy metal ions (such as Cu^2+^, Hg^2+^, Cr^3+^, Ni^2+^) (Figs 4a and S16). When the Ag@Azo_TPE_-CMP was treated in the ethanol-water solutions of corresponding metal salts with different concentrations, which can quenched the fluorescence of the Ag@Azo_TPE_-CMP. Cu^2+^, Cr^3+^, Hg^2+^, Ni^2+^ can quench the degree of fluorescence up to 99%, 97%, 96%, and 93%, respectively. Remarkably, as the concentration of Cu^2+^ decreased to 10^−6^ and 10^−7^ M, over 52% of the fluorescence was quenched. Even when the Cu^2+^ concentration was decreased to 10^−9^ M, the degree of fluorescence quenching was as high as 35% (Fig. 4b). The Ag@Azo_TPE_-CMP with such a high sensitivity for reporting Cu^2+^ outperform other Cu^2+^ chemo-sensors thus far reported^35–38^. Then, we further investigated the scope of ion species that can be sensed (Fig. 4a). Compared to Cu^2+^ ions, the Ag@Azo_TPE_-CMP exhibited low sensitivity and torpid response to other metal ions, such as Zn^2+^, Na^+^, Ba^2+^, and Ca^2+^, *etc*. These results clearly demonstrate that the Ag@Azo_TPE_-CMP are responsive to transition metal ions, such as Cu^2+^, Hg^2+^, Cr^3+^, Ni^2+^ (Fig. 4a). Besides the high sensitive of Ag@Azo_TPE_-CMP toward Cu^2+^, the anti-interference ability of the sensor is vitally important. Therefore, the emission spectra of Ag@Azo_TPE_-CMP dispersed in ethanol-water solutions containing three equal concentrations (10^−2^ M) of each metal ion (1: Zn^2+^, Ba^2+^ and Na^+^, Fig. 4c; 2: Al^3+^, Mn^2+^ and Ca^2+^, Fig. S17a; 3: La^3+^, Mg^2+^, Co^2+^, Fig. S17b) and subsequent addition of Cu^2+^ (10^−3^ M) have been monitored. It is apparent that the effective fluorescence quenching could occur upon adding 10^−3^ M Cu^2+^ into the parallel tests (Figs 4c and S17). These results indicate that Ag@Azo_TPE_-CMP possess outstanding anti-interference ability, sensitivity in the detection of Cu^2+^ even in the complicated system.

## Conclusions

In summary, two azo-linked conjugated microporous polymers have been successfully developed using a facile diazo-coupling reaction. Due to the metal silver effect, the Ag^+^ loading CMP showed the I_2_ uptake is 1.87-times than that of Azo_TPE_-CMP. In addition, Ag@Azo_TPE_-CMP displays outstanding performance for the detection of heavy ions such as Cu^2+^, Hg^2+^, Cr^3+^, Ni^2+^. Particularly, compared to other metal cations, Ag@AzoTPE-CMP shows more effective in the detection of Cu^2+^. These results clearly demonstrated that there is a wealth of opportunity for producing novel absorbent materials with enhanced iodine capture capacity, remove of heavy ions, and expanded the scope of applications.

### Synthesis of Azo_TPE_-CMP

Taking the preparation of Azo_TPE_-CMP as an example, the diazo-coupling reaction was carried out in two steps. Firstly, 4-(1, 2, 2-tris(4-aminophenyl)vinyl)benzenamine (1.5 mmol) was loaded in a 250 mL flask charged with 100 ml of deionized water, and 0.7 mL of concentrated hydrochloric acid. After stirred for 15 min at 0–5 °C, the mixture was added with 30 mL of aqueous solution of sodium nitrite (3.1 mmol) and stirred for 25 min to make amino groups be completely converted to diazonium salts. Subsequently, the mixture was neutralized with dilute solution of Na_2_CO_3_, and then mixed with 30 mL of aqueous solution of *m*-trihydroxybenzene (2 mmol) and Na_2_CO_3_ (3 mmol) at 0–5 °C. After 12 h solid sample was separated from the reaction solution by filtration, and washed by the solvents in the order: water, methanol, and THF, respectively. Followed by freeze drying, polymer sample was obtained in a high (78% yield)^39^.

### Synthesis of Ag@AzoTPE-CMP

Preparation of Ag nanoparticles in Azo_TPE_-CMP. A piece of Azo_TPE_-CMP (50 mg) was placed in 20 mL of aqueous solution 1 M NaOH to exchange the protons of -OH groups with Na^+^. After 3 h the Na^+^ exchanged Azo_TPE_-CMP was collected through filtration and washed with H_2_O. The wet Na^+^**@**Azo_TPE_-CMP was placed in 20 mL of H_2_O resulting in pH = 10. In that system, 100 mg of AgNO_3_ were added and allowed to react overnight. The collected monolithic piece was washed extensively with H_2_O, soaked in ethanol to exchange the H_2_O, and dried again with supercritical CO_2_. The final grey-white powder product was collected Ag@Azo_TPE_-CMP (200 mg)^40^.

## Electronic supplementary material

Supplementary Information
