# Supplementary material for: Ag+ doped into azo-linked conjugated microporous polymer for volatile iodine capture and detection of heavy metal ions
Source: Sci Rep. 2018 Sep 19;8:14072. doi: 10.1038/s41598-018-32383-5 (PMC6145922; doi:10.1038/s41598-018-32383-5)
Supplement: Supplementary file 1 — Supplementary Information [file 41598_2018_32383_MOESM1_ESM.doc]

**Supporting Information**

**Ag^+^ doped into azo-linked conjugated microporous polymer for volatile iodine capture and detection of heavy metal ions**

Minghan Liu,^a^ Chan Yao,^a^ Chunbo Liu^c^ and Yanhong Xu*^a,b^

Corresponding Author:

Professor Yan-Hong Xu

^a^Key Laboratory of Preparation and Applications of Environmental Friendly Materials (Jilin Normal University), Ministry of Education, Changchun, 130103, China

^b^Key Laboratory of Functional Materials Physics and Chemistry of the Ministry of Education, Jilin Normal University, Siping 136000, China

Email: [xuyh198@163.com](mailto:xuyh@jlnu.edu.cn)

*^c^Institute of Green Chemistry & Chemical Technology, Jiangsu University, Zhenjiang 212013, China*

**Contents**

**Section A. Materials and methods**

**Section B. Synthetic procedures**

**Section C. The solid-state ^13^C CP-MAS NMR**

**Section D. Powder X-ray diffraction patterns**

**Section E. FT-IR spectral profiles**

**Section F. TGA curves**

**Section G. HR-TEM images**

**Section H. UV/Vis spectra and photoluminescence spectra**

**Section I. Iodine capture analyses**

**Section J. Corresponding removal of heavy ions**

**Section K. Supporting references**

**Section A. Materials and methods**

*m*-Trihydroxybenzene was purchased from Aladdin. Sodium nitrite and anhydrous sodium carbonate were purchased from Energy Chemical. All the solvents used were purchased from Aladdin.

^1^H NMR spectra were recorded on Bruker Advance III models HD 400NMR spectrometers, where chemical shifts (*δ* in ppm) were determined with a residual proton of the solvent as standard. Fourier transform Infrared (FT-IR) spectra were recorded on a Perkin-elmer model FT-IR-frontier infrared spectrometer. The solution UV-visible analyzer was used for shimadzu UV-3600. Photoluminescence spectra were recorded on a shimadzu F-4600 spectrometer (JAPAN) spectrofluorometer. Solid-state ^13^C CP/MAS NMR measurements were recorded using a Bruker AVANCE III 400 WB spectrometer at a MAS rate of 5 kHz and a CP contact time of 2 ms. Field-emission scanning electron microscopy (FE-SEM) images were performed on a JEOL model JSM-6700 operating at an accelerating voltage of 5.0 kV. The samples were prepared for SEM by drop-casting a tetrahydrofuran suspension onto mica substrate and then coated with gold, while samples for TEM observations prepared on copper grids. High-resolution transmission electron microscopy (HR-TEM) images were obtained on a JEOL model JEM-3200 microscopy. Powder X-ray diffraction (PXRD) data were recorded on a Rigaku model RINT Ultima III diffractometer by depositing powder on glass substrate, from 2*θ* = 1.5° up to 60° with 0.02° increment. X-ray photoelectron spectra (XPS) were recorded on an ESCALAB250Xi electron spectrometer (Thermo Fisher Scientific Inc., Waltham, MA, USA). The elemental analysis was carried out on a EuroEA-3000. TGA analysis was carried out using a Q5000IR analyser (TA Instruments) with an automated vertical overhead thermobalance. Before measurement, the samples were heated at a rate of 5 °C min^-1^ under a nitrogen atmosphere.

Nitrogen sorption isotherms were measured at 77 K with ASIQ (iQ-2) volumetric adsorption analyzer. Before measurement, the samples were degassed in vacuum at 150 °C for more than 10 h. The Brunauer-Emmett-Teller (BET) method was utilized to calculate the specific surface areas and pore volume. The nonlocal density functional theory (NLDFT) method was applied for the estimation of pore size and pore size distribution.

**Section B. Synthetic procedures**

**Synthesis of 4-(1,2,2-tris(4-aminophenyl)vinyl)benzenamine^S1^**

Benzophenone (300 mg, 1.41 mmol) and Sn powder (400 mg, 3.37 mmol) were added to a 50 mL two-necked flask at room temperature under normal pressure and 10 mL of potassium bromide (48%) and 10 mL of concentrated hydrochloric acid into the beaker mixed evenly, while stirring slowly mixing the mixed acid into the two-necked flask, stirring heating 5 h. 100 mL of saturated sodium hydroxide solution was placed and allowed to stand for cooling. The product cooled in two flasks was slowly added with stirring to the saturated sodium hydroxide beaker and stirred for 1 h to rapidly filter to give a yellowish green solid (54.6% yield). ^1^H NMR (400 MHz, DMSO-d_6_) δ 6.56 (d, *J* = 8.2 3 Hz, 8H), 6.25 (d, *J* = 8.2 Hz, 8H), 4.86 (s, 8H).

**Section C. The solid-state ^13^C CP-MAS NMR**

**
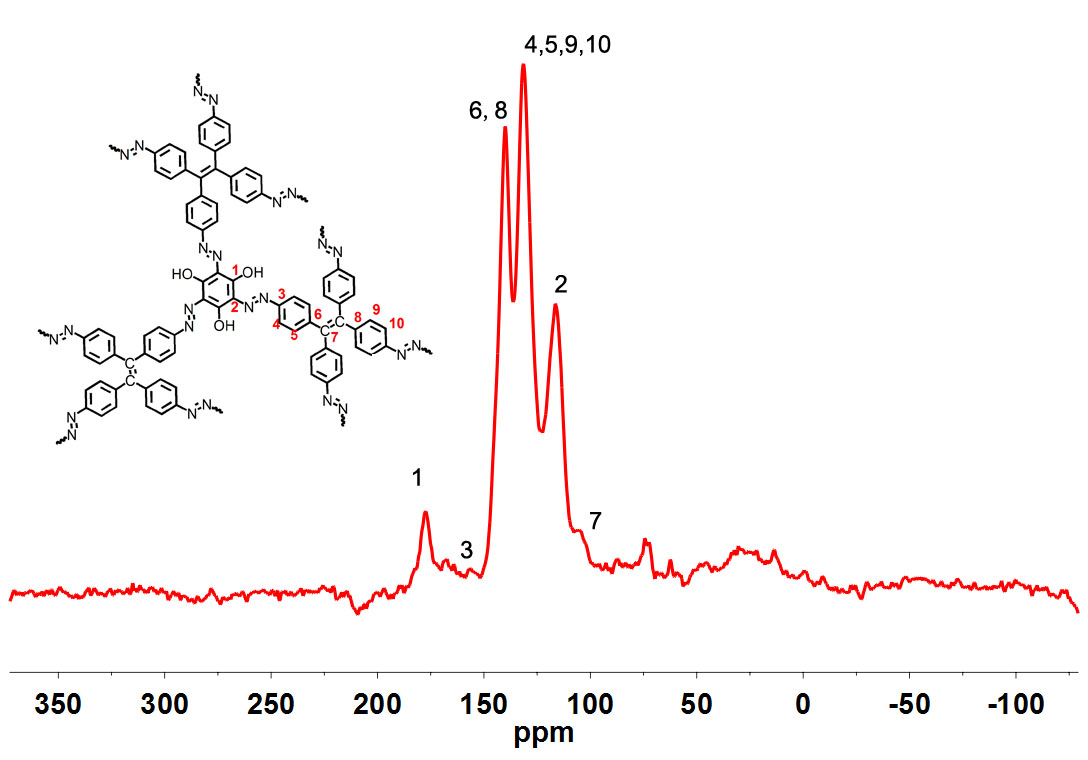
**

**Figure S1.** The solid-state ^13^C CP-MAS NMR of Azo_TPE_-CMP.

**Section D. Powder X-ray diffraction patterns**


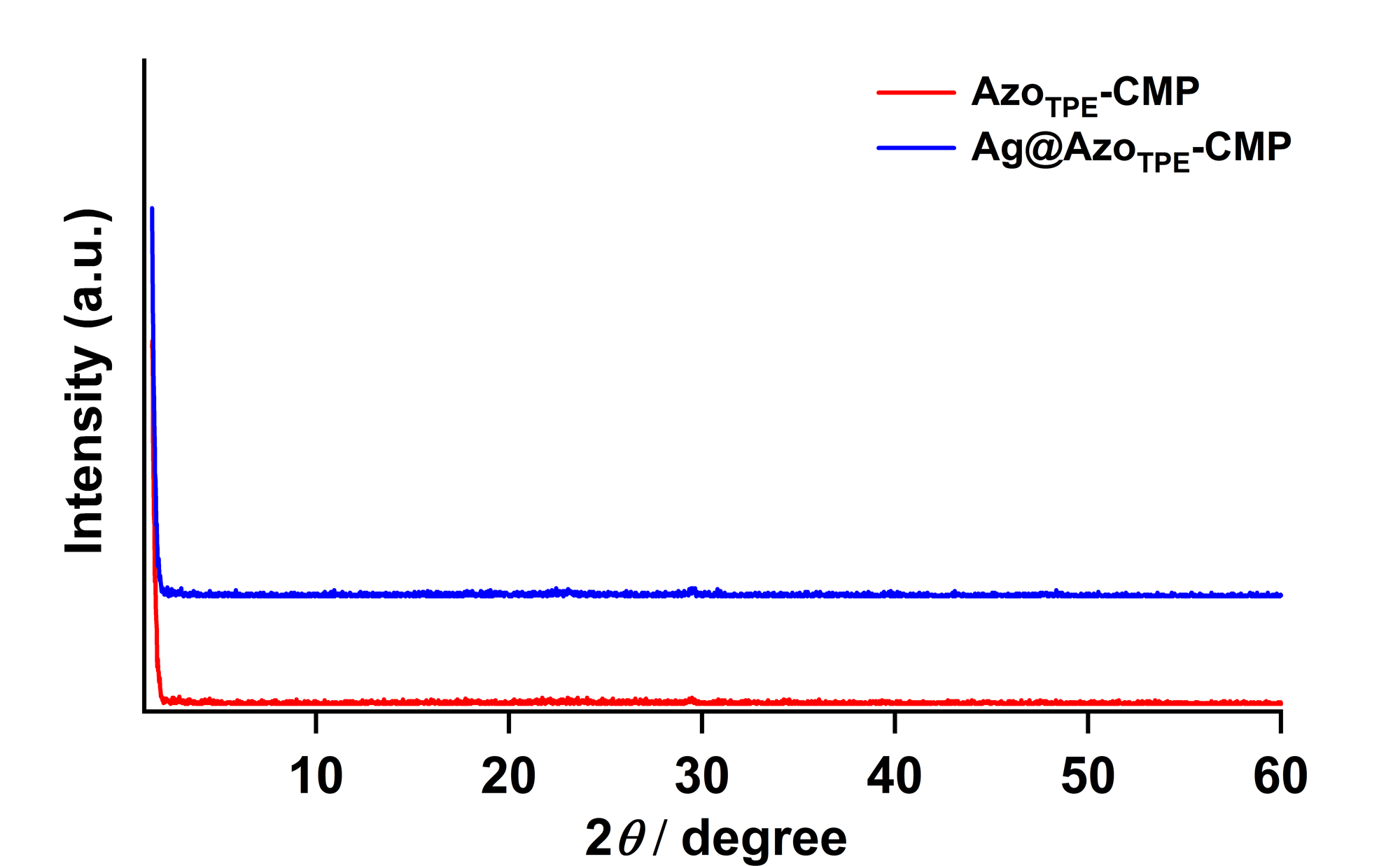


**Figure S2.** Powder X-ray diffraction profiles of Azo_TPE_-CMP and Ag@Azo_TPE_-CMP.

**Section E. FT-IR spectral profiles**

**
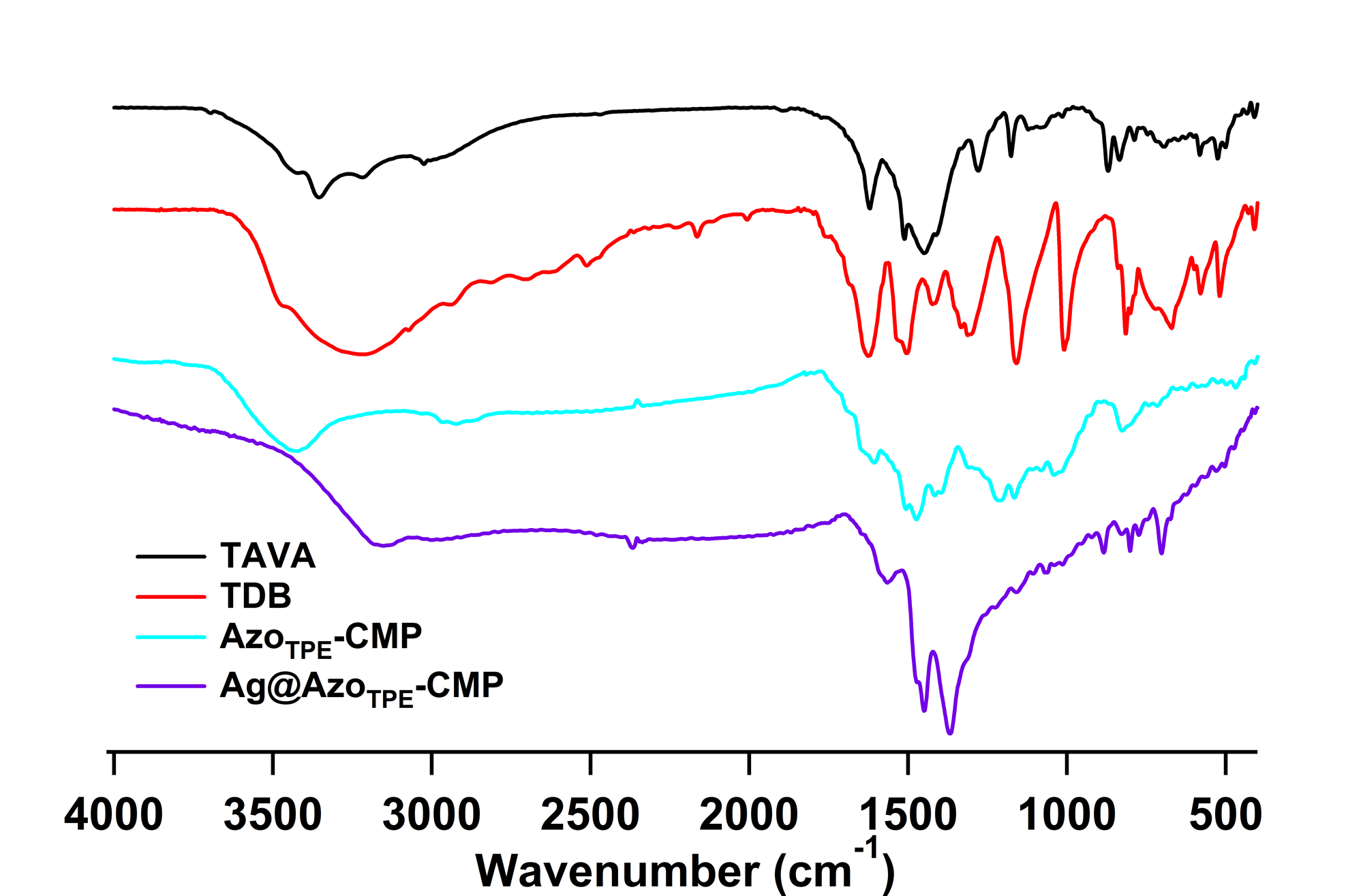
**

**Figure S3.** FT-IR spectra of Azo_TPE_-CMP and Ag@Azo_TPE_-CMP.

**Section F. TGA curves**

**
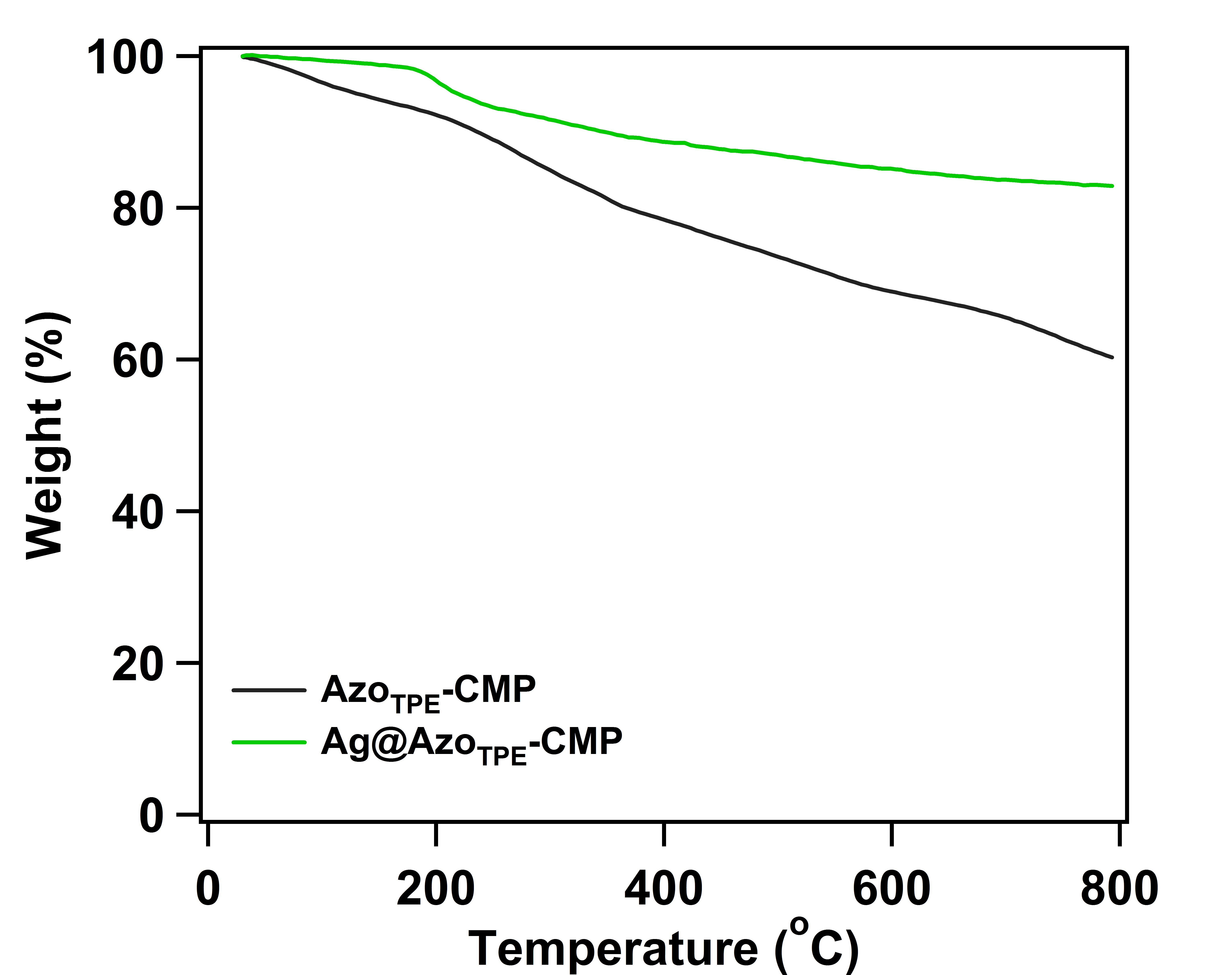
**

**Figure S4.** TGA curves of Azo_TPE_-CMP and Ag@Azo_TPE_-CMP.

**Section G. HR-TEM images**

**
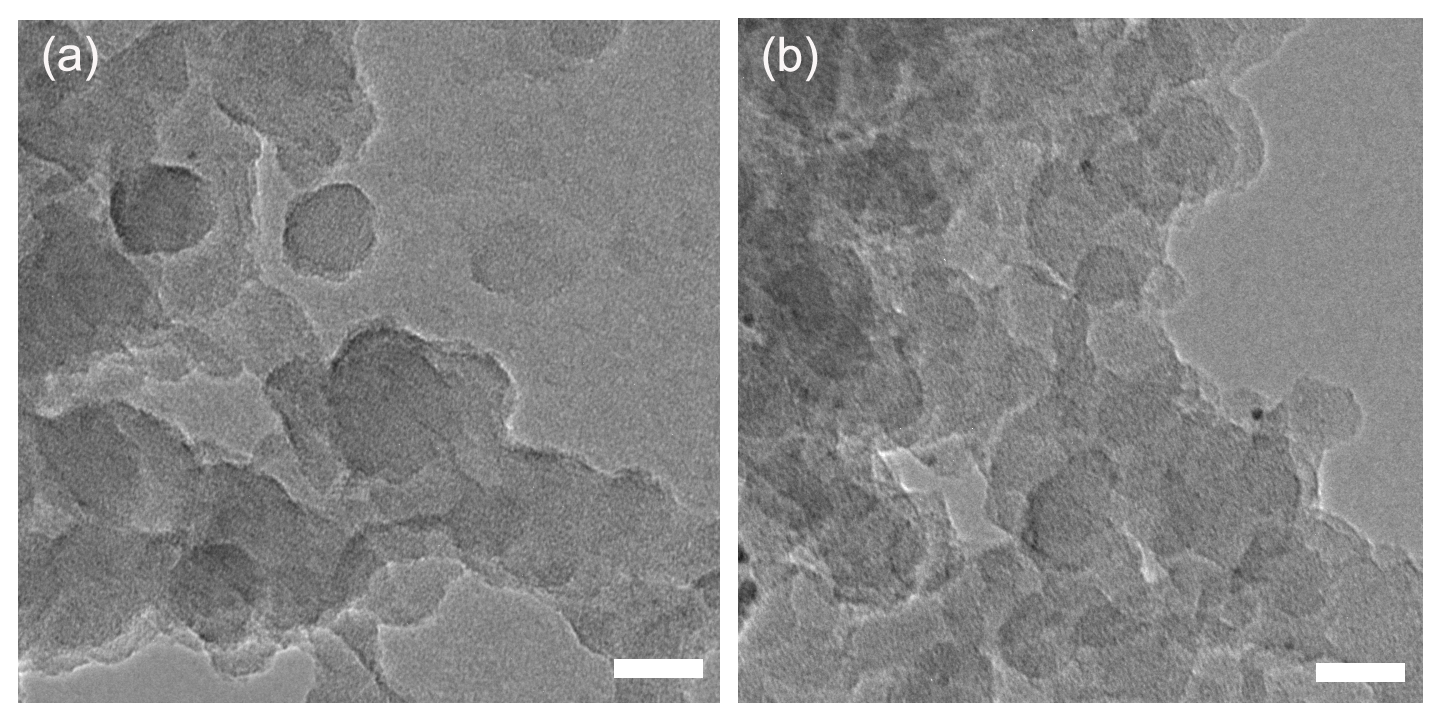
**

**Figure S5.** HR-TEM images of (a) Azo_TPE_-CMP and (b) Ag@Azo_TPE_-CMP (scale bar 10 nm), respectively.

**Section H. UV/Vis spectra and photoluminescence spectra**


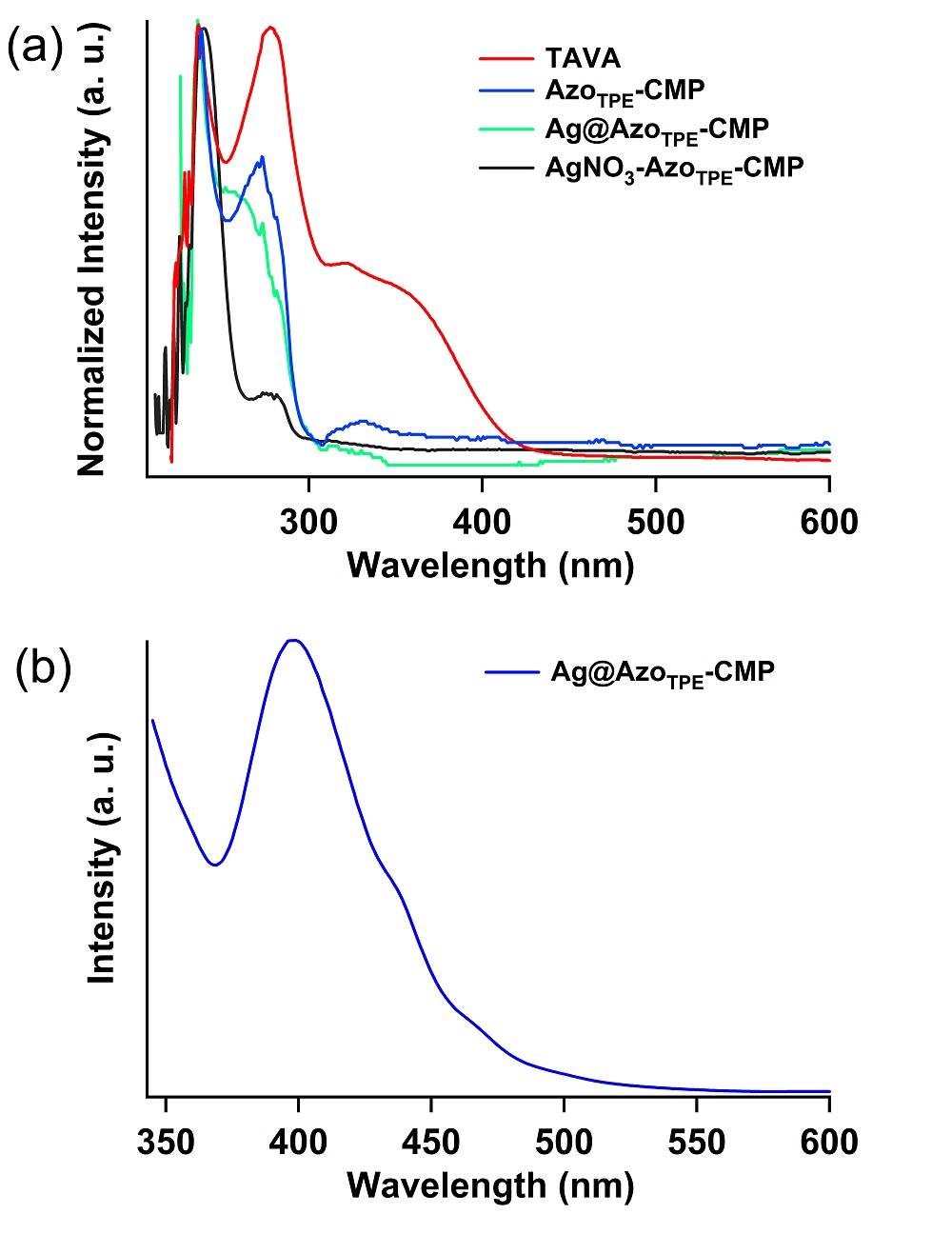


**Figure S6.** (a) UV-vis spectra of azo-linked polymers, and (b) photoluminescence spectra of Ag@AzoTPE-CMP.


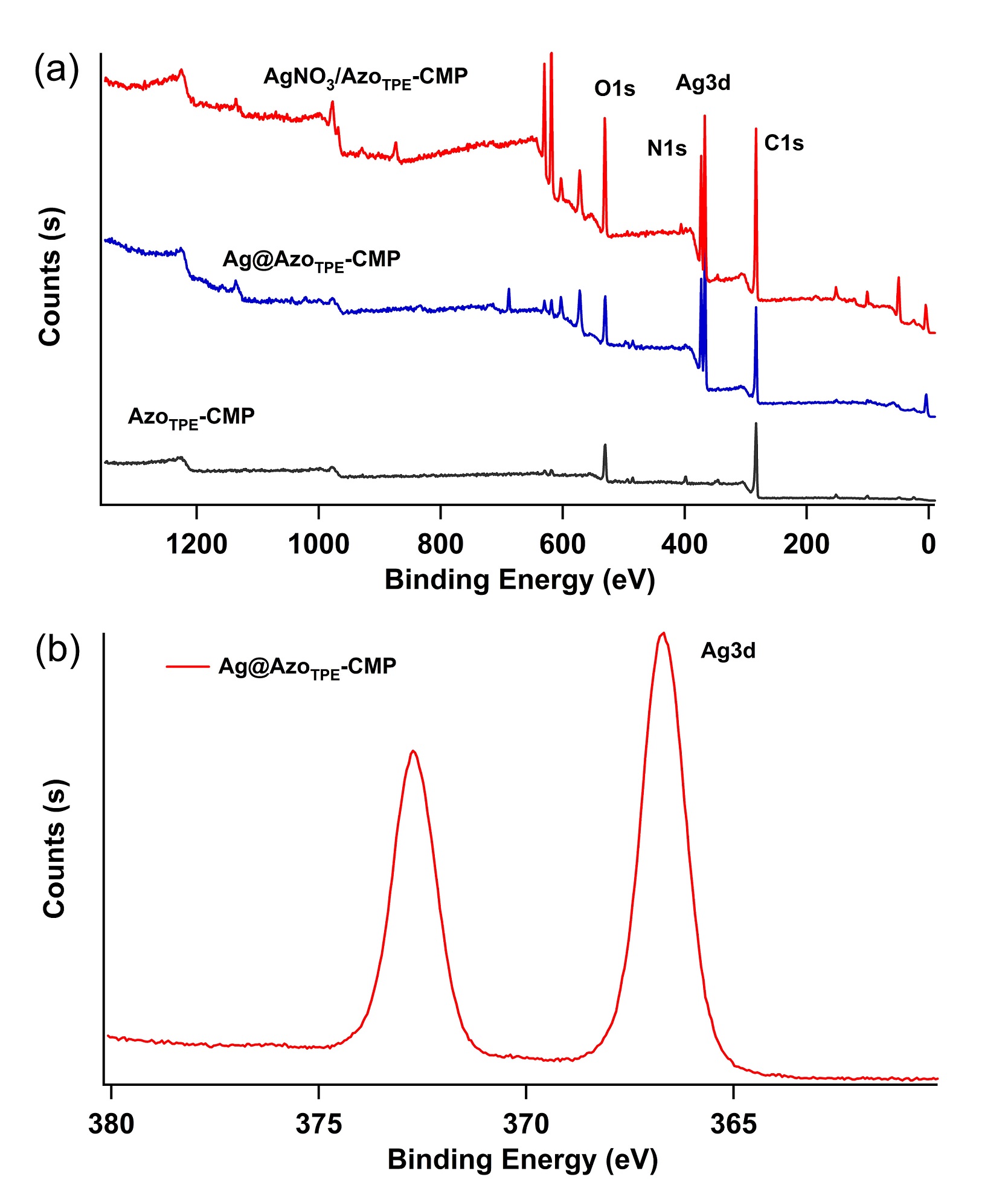


**Figure S7.** XPS survey spectra of the two azo-linked CMPs.

**Section I. Iodine capture analyses**

**(1) XPS spectra**


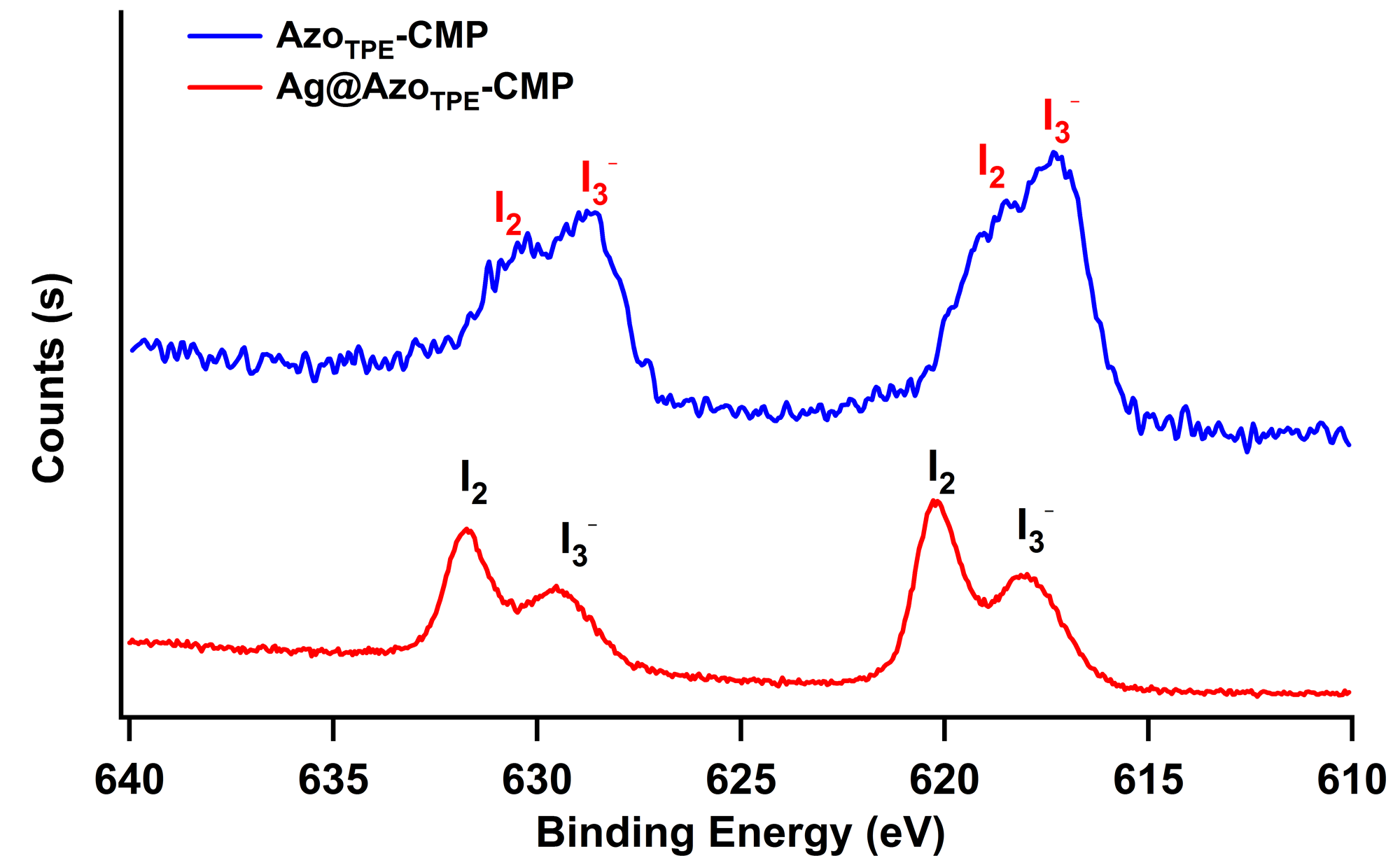


**Figure S8.** XPS spectra of the azo-linked CMPs after iodine capture.

**(2) Recyclability for iodine uptake**


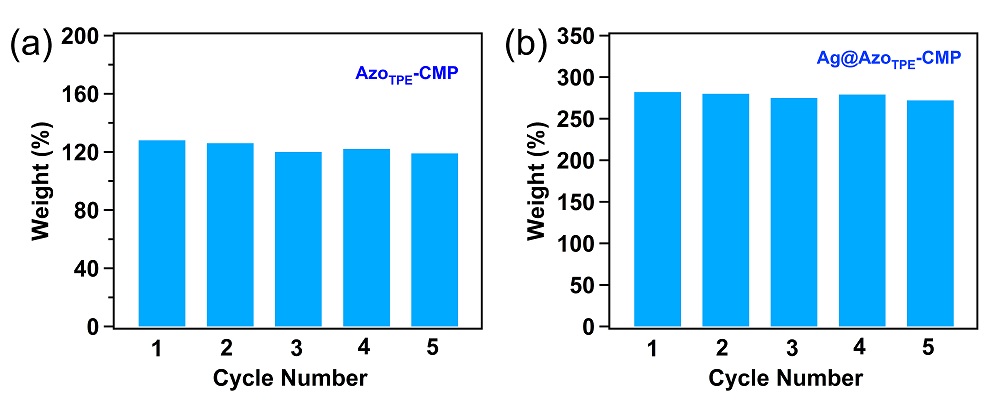


**Figure S9.** Reusability of the azo-linked CMPs for iodine adsorption by vapor sublimation.


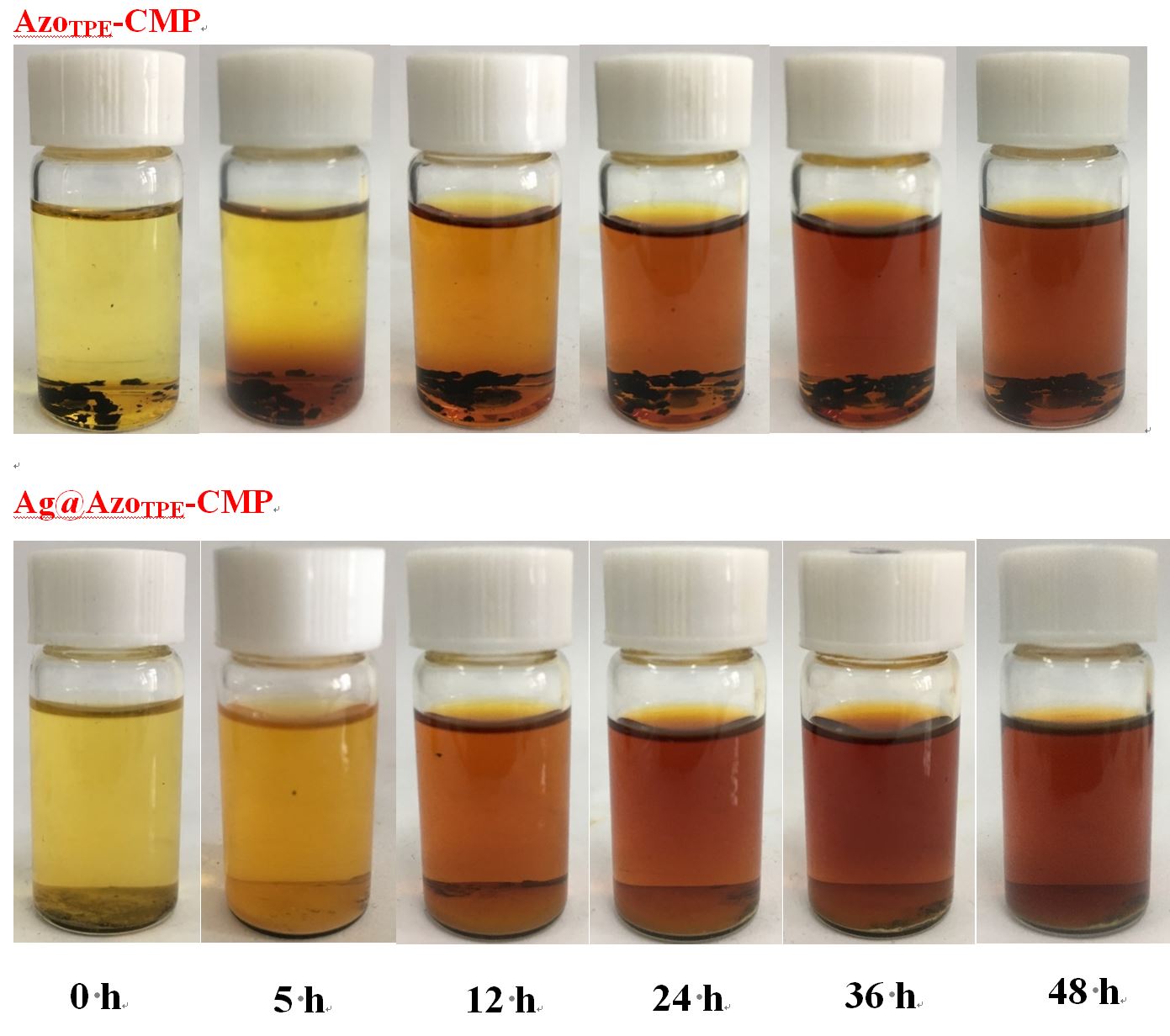


**Figure S10.** The figures of the released I_2_ guests from the azo-linked networks.

**
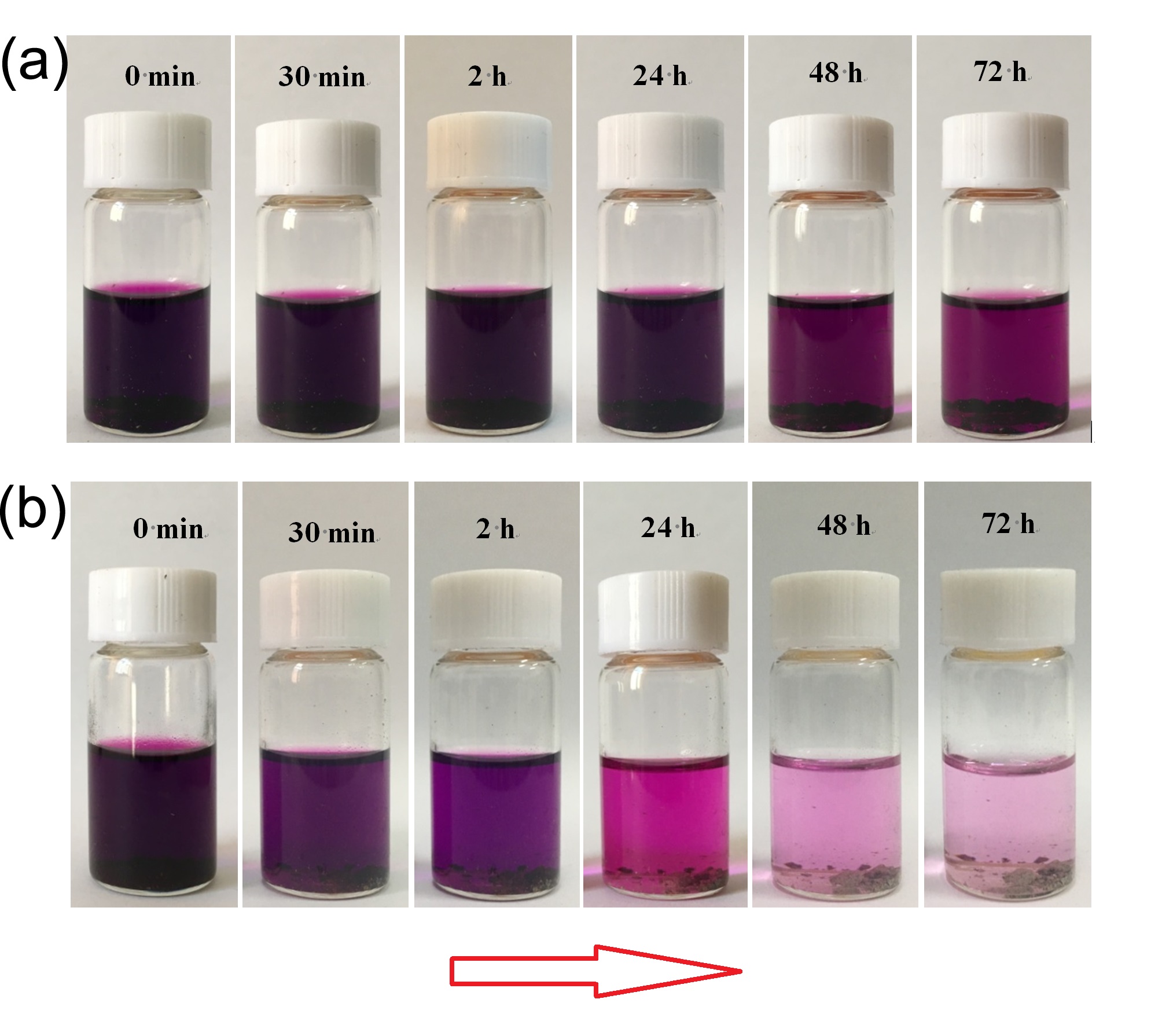
**

**Figure S11.** The photographs show the different iodine adsorption rates of (a) Azo_TPE_- CMPs, and (b) Ag@Azo_TPE_- CMPs.


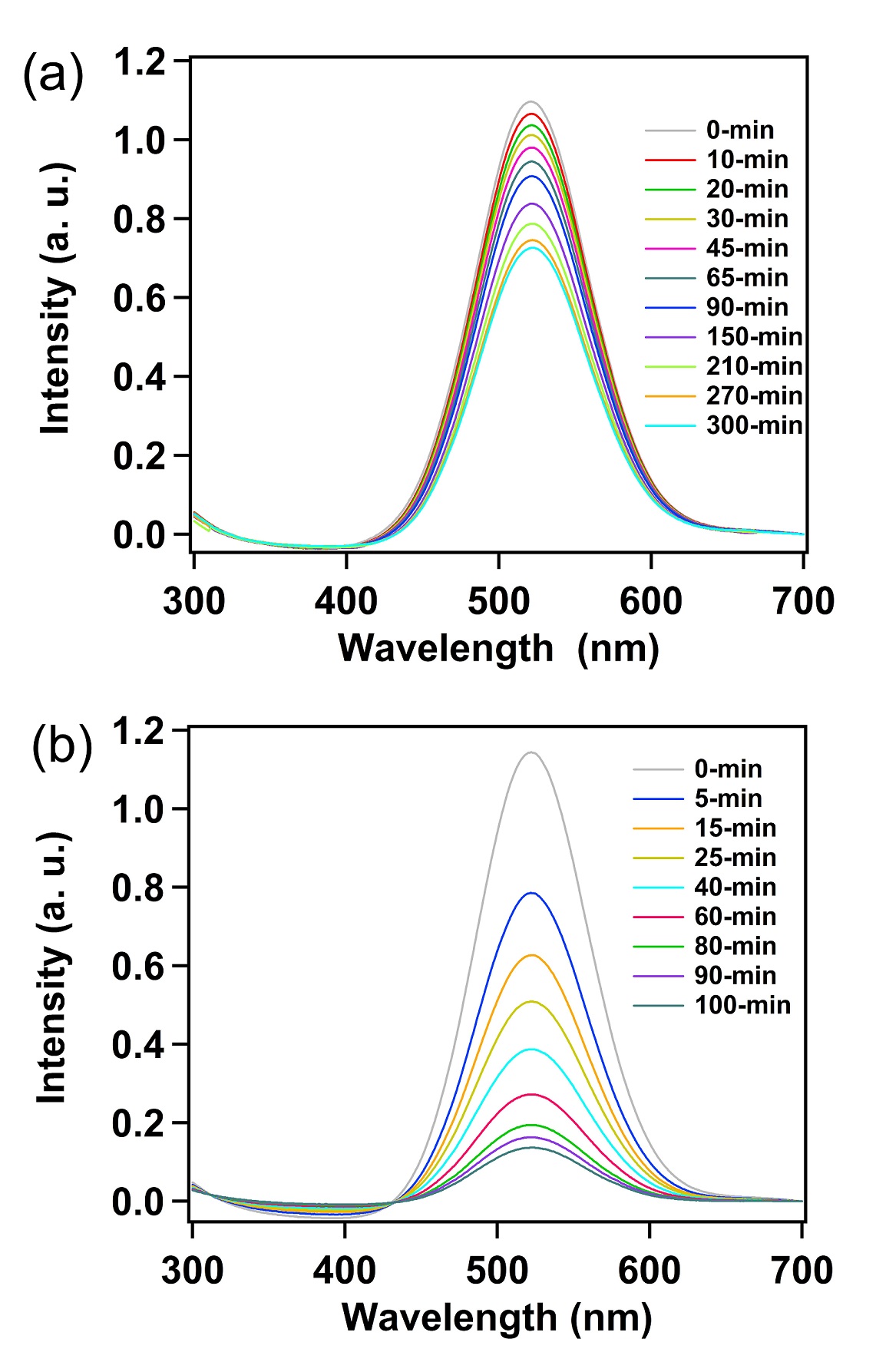


**Figure S12.** UV/Vis spectra upon immersion of 30 mg azo-linked CMP in cyclohexane solution of I_2_ (10^–2^ M), (a) Azo_TPE_-CMP and (b) Ag@Azo_TPE_-CMP. All experiments were performed at ambient temperature and pressure.

**(3) Sorption kinetic studies**

**The linear form of the pseudo-first-order kinetic model:**


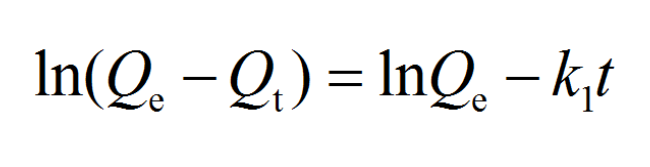


Where *Q*_t_ and *Q*_e_ are the mass percent of iodine adsorbed at time t and equilibrium (%), *k*_1_ is the pseudo-first-order rate constant of adsorption process (h^-1^).

**The linear form of the pseudo-second-order kinetic model:**


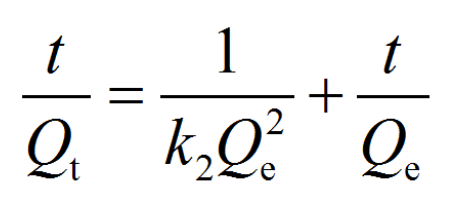


Where *Q*_t_ and *Q*_e_ are the mass percent of iodine adsorbed at time t and equilibrium (%), *k*_2_ is the pseudo-second-order rate constant of adsorption process ((% h)^-1^).


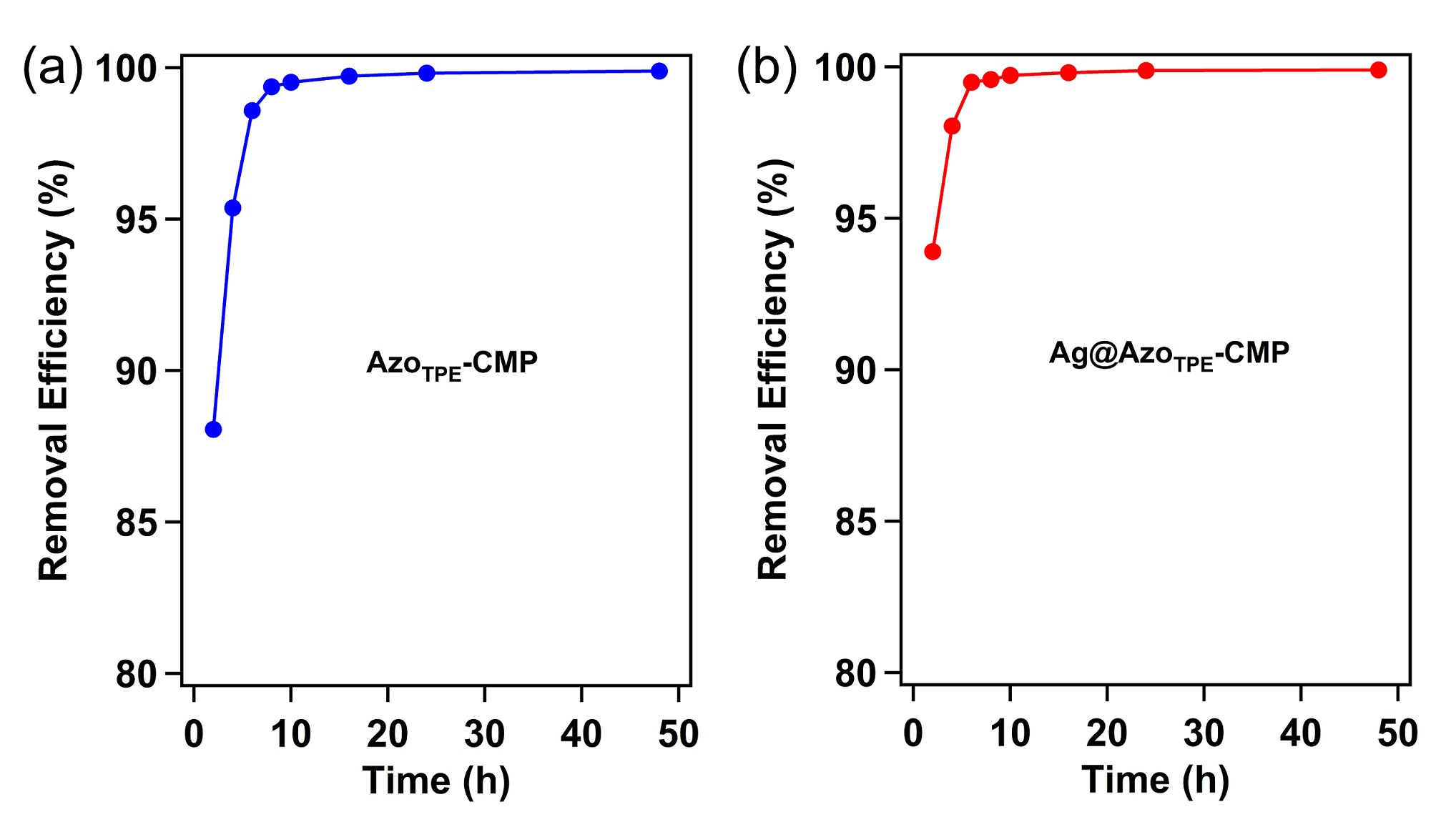


**Figure S13.** Kinetic studies of iodine adsorption by immersing the azo-linked CMPs in hexane solution (4 mg mL^-1^).

**
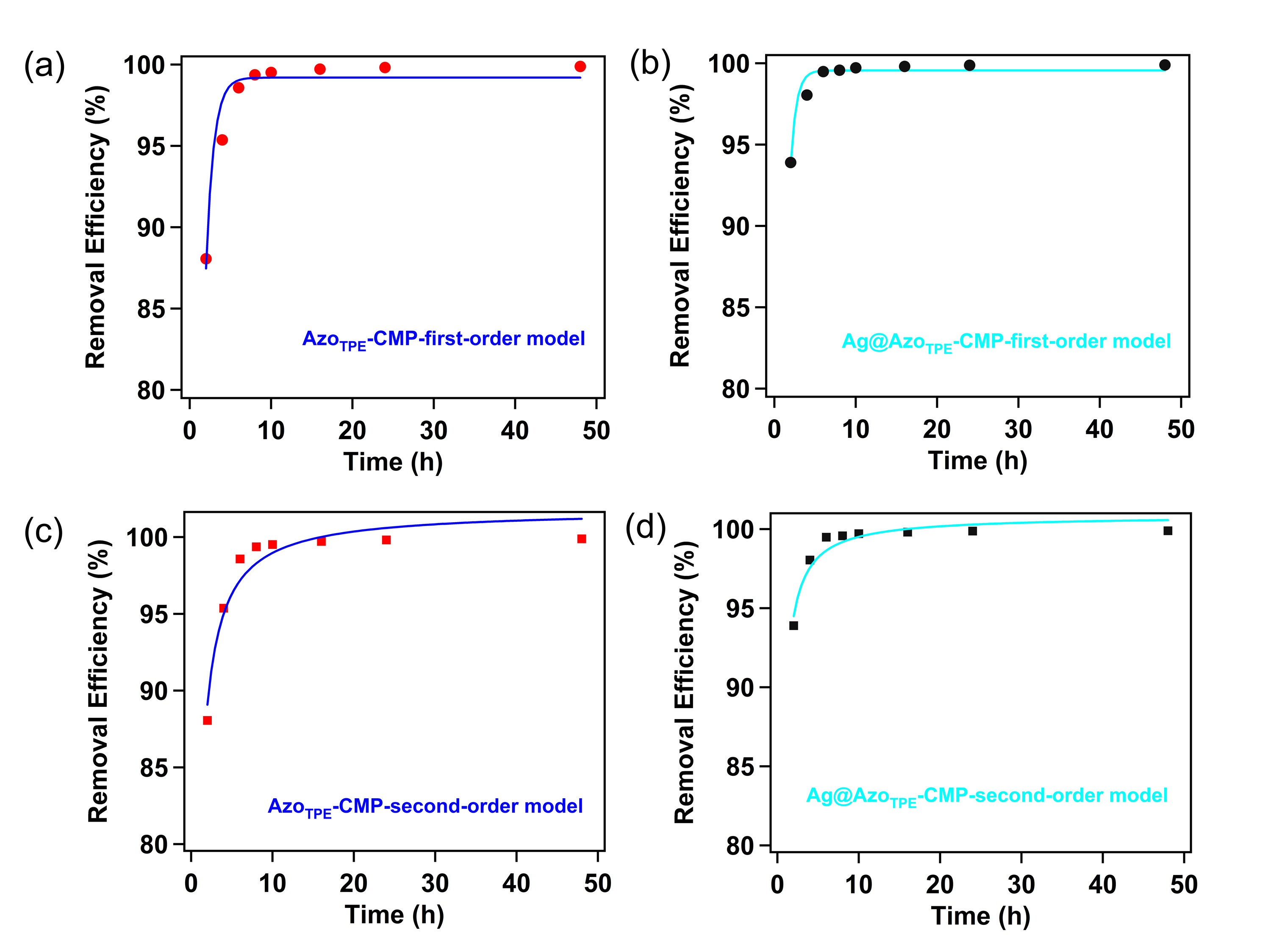
**

**Figure S14.** (a-b) The iodine adsorption kinetic was analyzed by the pseudo-first-order model; (c-d) the iodine adsorption kinetic was analyzed by the pseudo-second-order model. Initial concentration of iodine solution: 4 mg mL^-1^.

**Table S1. Porosity properties for the polymers.**

| Polymers | *S*_BET_^a^  /m^2^ g^-1^ | *S*_micro_^b^  /m^2^ g^-1^ | V_total_^c^  /cm^3^ g^-1^ | V_micro_  /cm^3^ g^-1^ |
| --- | --- | --- | --- | --- |
| **LCMP-1** | 366.33 | 152.42 | 1.0721 | 0.9433 |
| **Ag@LCMP-1** | 47.325 | 0.592 | 0.1099 | 0.1099 |

*^a^*Brunauer-Emmett-Teller surface area. *^b^*Total pore volume determined from the N_2_ isotherm at P/P_0_ =0.995. *^c^*Micro-pore volume determined from the N_2_ isotherm at P/P_0_ = 0.050.

**Table S2. Parameters of the different isotherm models extracted from the pseudo-first-order model and the pseudo-second-order model for the azo-linked CMPs.**

| **Adsorbent** | **Pseudo-first-order** | | | **Pseudo-second-order** | | |
| --- | --- | --- | --- | --- | --- | --- |
|  | *k*_1_ (1/h) | *Q*_e_ (%) | *R*^2^ | *k*_2_ (1/h) | *Q*_e_ (%) | *R*^2^ |
| **Azo_TPE_-CMP** | 1.0670 | 0.9920 | 0.9232 | 0.0344 | 1.0180 | 0.9309 |
| **Ag@Azo_TPE_-CMP** | 1.4217 | 0.9956 | 0.9336 | 0.0738 | 1.0086 | 0.9156 |

**(4) Adsorption Isotherm Models
The Langmuir isotherm model:**


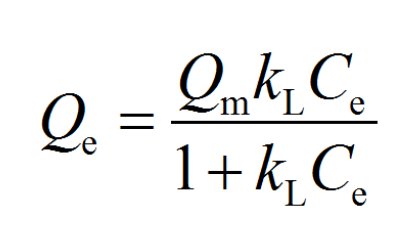


**The Freündlich isotherm model:**


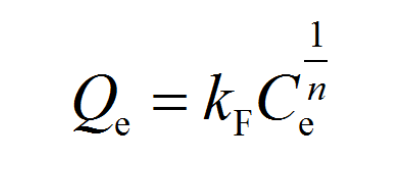


Where *k*_L_ (mg^-1^) and *Q*_m_ (mg g^-1^) are the Langmuir isotherm constants; *k*_F_ (mg^-1^) is the Freündlich isotherm constant; *C*_e_ is the concentration at equilibrium (mg mL^-1^), *Q*_e_ is the amount of iodine adsorbed at equilibrium (mg g^-1^).

**Table S3. Parameters of two simulation models extracted from experimental adsorption isotherms data for the azo-linked CMPs.**

| **Adsorbent** | **Langmuir isotherm** | | | **Freundlich isotherm** | | |
| --- | --- | --- | --- | --- | --- | --- |
|  | Q_m_ (mg/g) | *k*_L_(l/mg) | *R*^2^ | *k*_F_ (l/mg) | n | *R*^2^ |
| **Azo_TPE_-CMP** | 1991 | 0.0865 | 0.9720 | 218.537 | 1.632 | 0.9254 |
| **Ag@Azo_TPE_-CMP** | 2598 | 0.0721 | 0.9580 | 233.629 | 1.526 | 0.9098 |

**
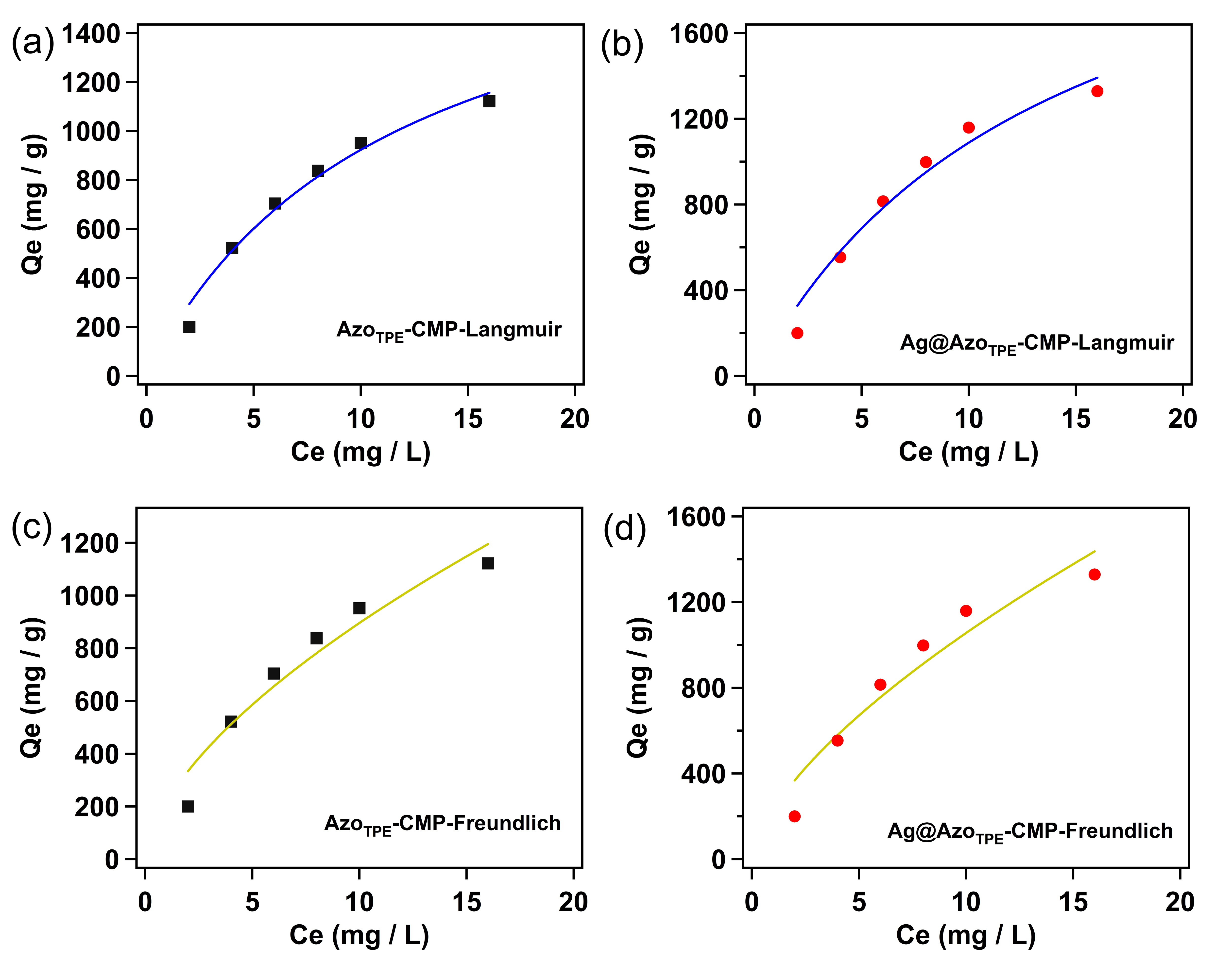
**

**Figure S15.** Adsorption isotherm of the azo-linked CMPs for iodine (15 mg polymer was soaked in 3 mL of iodine solution at various concentrations for 48 h). (a-b) Fitting curve: Langmuir. (c-d). Fitting curve: Freundlich.


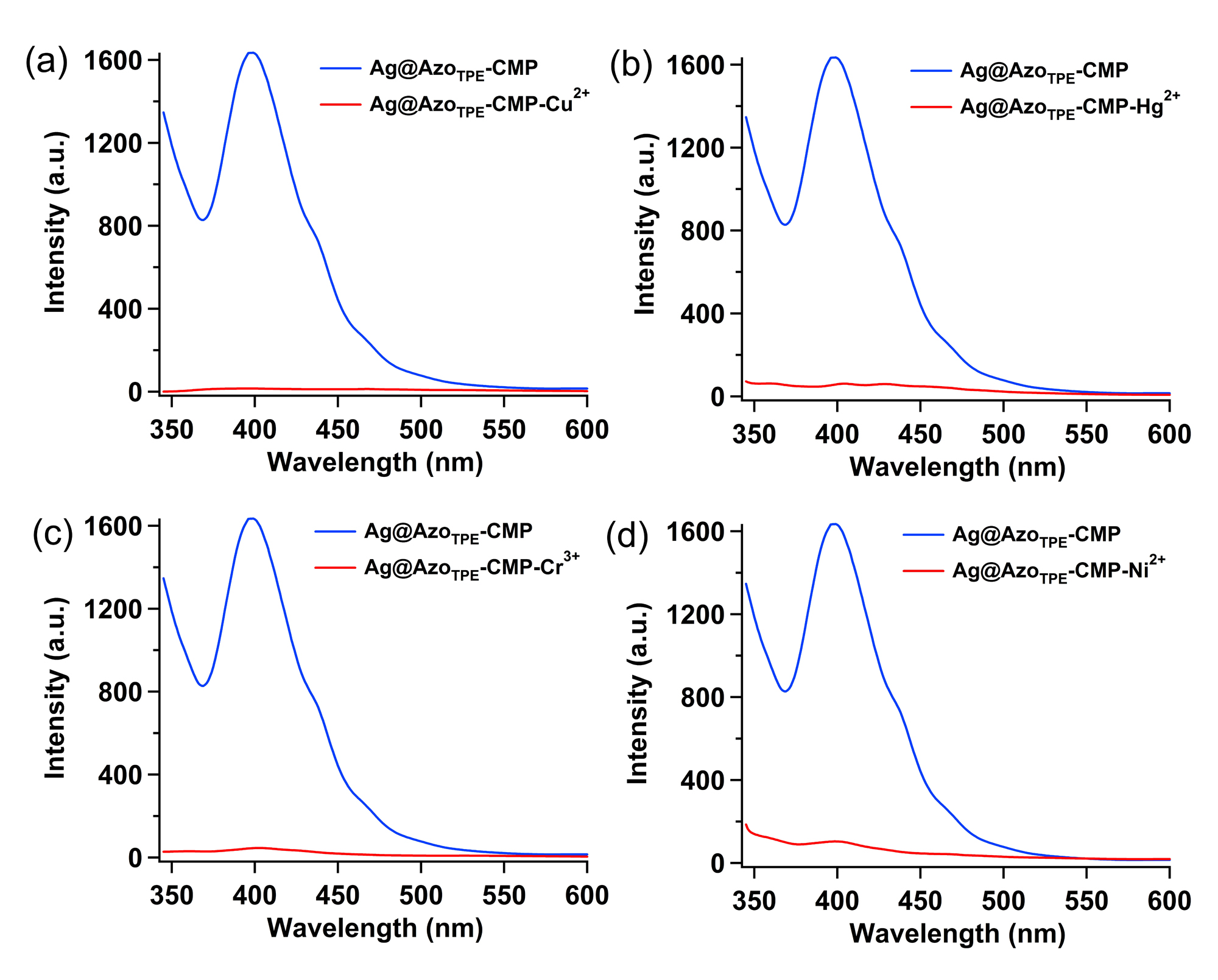


**Figure S16.** Degree of fluorescence quenching of the Ag@Azo_TPE_-CMP in ethanol-water solutions of different metal ions (10^-2^ M).

**Section J. Corresponding removal of heavy ions**

**
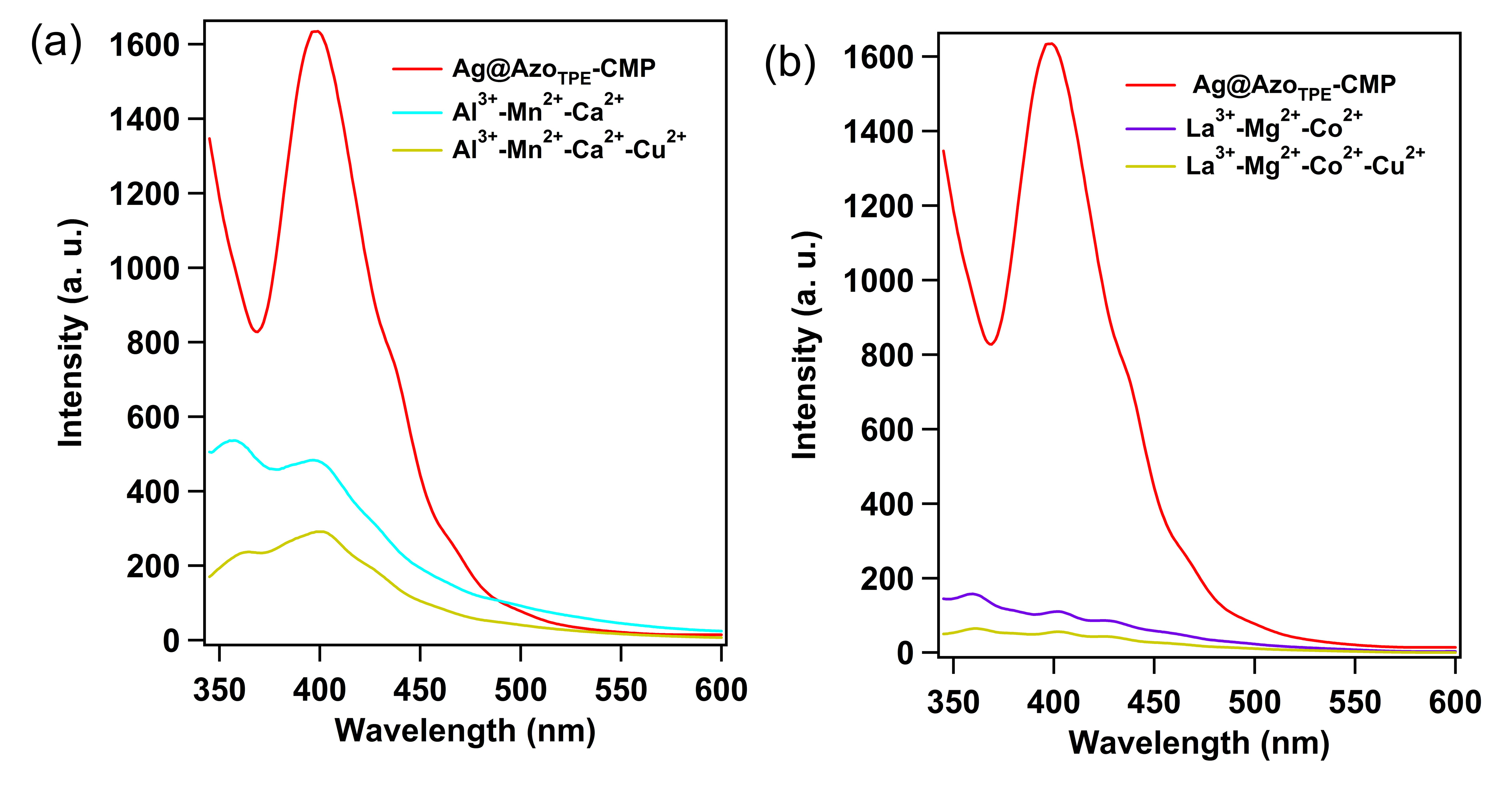
**

**Figure S17.** Degree of fluorescence quenching of Cu^2+^ (10^-3^ M) in ethanol solutions of different metal ions (10^-2^ M).

**Section K. Supporting references**

S1. Xu, S. Q.; Zhang, X.; Nie, C. B.; Pang, Z. F.; Xu, X. N.; Zhao, X.; ***Chem. Commun.***, **2015**, ***51***, 16417-16420.
